# Prevalence and Determinants of Significant Liver Fibrosis by Vibration-Controlled Transient Elastography in Thai Chronic Hepatitis B Patients

**DOI:** 10.1155/2018/4310102

**Published:** 2018-10-02

**Authors:** Apichet Sirinawasatien, Thanaya Techasirioangkun, Siriporn Thongsri

**Affiliations:** Division of Gastroenterology, Department of Medicine, Rajavithi Hospital, College of Medicine, Rangsit University, Bangkok, Thailand

## Abstract

**Aims:**

To describe the prevalence of significant liver fibrosis by ultrasound-based vibration-controlled transient elastography (VCTE; FibroScan®) and to identify the determinants of significant liver fibrosis in Thai chronic hepatitis B patients.

**Methods:**

A cross-sectional study of consecutive chronic hepatitis B patients performed VCTE and followed up at Rajavithi Hospital, Bangkok, Thailand, was conducted between 1 January, 2013, and 31 December, 2016. Liver fibrosis was defined as minimal (METAVIR F0-1) by VCTE < 7.2 kPa and significant (METAVIR F2-4) by VCTE ≥ 7.2 kPa. VCTE assessments and medical records were retrospectively reviewed. The prevalence and determinants of significant liver fibrosis were analyzed.

**Results:**

A total of 206 eligible patients were included; 120 patients (58.3%) were female. The mean age was 50 years (SD 12.4 years), and 32.5% had a body mass index ≥ 25. The prevalences of minimal (F 0-1) and significant fibrosis (F2-4) were 74.3% and 25.7%, respectively. The prevalence of hepatitis B e antigen negative (HBeAg -ve) was 83%. The median serum hepatitis B virus viral load was 4,340 IU/mL (range 20-271,883,036). Significant determinants of significant fibrosis (F2-4) were male gender (aOR 3.24 [95%CI: 1.36-7.72]) and high aspartate transaminase (AST) level (aOR 5.71 [95%CI: 2.03-16.04]).

**Conclusion:**

Around one-quarter of the Thai patients with chronic viral hepatitis B had significant liver disease defined by VCTE, requiring further evaluation for specific treatment for hepatitis B virus. Determinants of significant liver fibrosis were male gender and high AST level.

## 1. Introduction

Hepatitis B virus (HBV) is among the common causes of cirrhosis and hepatocellular carcinoma worldwide, especially in the Asia-Pacific region, including Thailand [1]. Patients have a wide range of clinical manifestations from inactive hepatitis B surface antigen carriers with normal histology to advanced liver fibrosis. The management of chronic hepatitis B infection is thus complex and based upon multiple factors, e.g., HBV viral load, hepatitis B e antigen (HBeAg) status, the presence or absence of liver inflammation, fibrosis, and cirrhosis. Patients with persistently high viremia with moderate to severe fibrosis (i.e. a METAVIR score ≥ F2) or cirrhosis should have antiviral treatment initiated with either a finite duration of pegylated interferon or long-term treatment with nucleos(t)ide analogues [2–4].

Liver biopsy remains the gold standard for evaluating the degree of liver fibrosis. It has, however, several disadvantages, including invasiveness, associations with rare serious complications, and susceptibility to sampling variation due to sampling only a small portion of the liver parenchyma. Many radiologic methods for staging liver fibrosis, including ultrasound-based and vibration-controlled transient elastography (VCTE), have recently been increasingly used in clinical practice instead of liver biopsy. VCTE is quick, reproducible, and painless, and it examines a large portion of liver parenchyma, thereby reducing sampling error. VCTE is the most studied radiologic method and is reliable for staging liver fibrosis in HBV- or hepatitis C virus (HCV)-infected patients, and other liver diseases [5–11]. Recent guidelines from the European Association for the Study of Liver Diseases and the Latin American Association for the Study of the Liver recommends VCTE as a valid noninvasive test for assessing liver fibrosis in HBV- and HCV-infected patients with a >90% negative predictive value for ruling out cirrhosis [12].

There have been few studies about the prevalence of significant liver fibrosis in chronic viral hepatitis B patients in Thailand. It is also important to identify the predictors of underlying significant liver fibrosis to select the patients who may benefit from early initiation of antiviral treatment. We thus aimed to describe the prevalence of significant liver fibrosis by noninvasive test (VCTE) in Thai chronic viral hepatitis B patients and also to identify the determinants of significant liver fibrosis.

## 2. Materials and Methods

### 2.1. Patients

This cross-sectional study was conducted by reviewing the medical records of all chronic hepatitis B patients who has VCTE performed from 1 January, 2013, to 31 December, 2016, at the outpatient clinic of the Department of Medicine at Rajavithi Hospital, a tertiary referral center in Bangkok, Thailand. Patients older than 18 years with chronic hepatitis B infection defined as having a positive HBs antigen for 6 months or more were included. None received antiviral treatment, namely nucleos(t)ide analogues or pegylated interferon, before VCTE was performed.

The exclusion criteria were (1) the presence of HCV or human immunodeficiency virus coinfection, (2) a positive test for anti-nuclear antibody, anti-smooth muscle antibody or anti-mitochondrial antibody, (3) a significant elevation of liver enzymes (aminotransferases > 5-fold the upper limit of normal [ULN]), and (4) an invalid VCTE result. The study protocol was reviewed and approved by the ethics committee of Rajavithi Hospital (No. 110/2560).

### 2.2. Vibration-Controlled Transient Elastography

VCTE were performed by one experienced operator (Thanaya Techasirioangkun, RN), using a Fibroscan® device (Echosens, France). This device sends a shear wave through the liver. The velocity of the propagation of this wave is assessed as a measure of the elasticity, or the stiffness, of the liver. Higher liver stiffness causes the shear wave to move faster, and higher liver stiffness indicates the presence of fibrosis. The VCTE measurement technique used involves a piston vibrator placed in the 9th-11th intercostal space on the right lobe of the liver with the patient positioned supine with the right arm at maximal abduction. The velocity is measured in a region 25-65 mm below the skin surface with the standard adult M probe. The unit of measurement is kPa with a range from 2.5 to 75 kPa. A valid VCTE result requires at least 10 successful measurements and an interquartile range (reflects variations among measurements) <30% of the median value [13, 14].

Factors influencing viscoelasticity of the liver may also contribute to increased liver stiffness such as the presence of severe hepatic inflammation [15] and nonfasting state [16]. Patients with significant elevation in liver enzymes (aminotransferases >5-fold the ULN) were thus excluded from the study, and VCTE was performed after the patient had fasted for at least 2 hours.

Stages of liver fibrosis were categorized according to the previous study by Marcellin et al. [8]. Liver stiffness scores of <7.2 kPa, ≥7.2 kPa, ≥8.1 kPa, and ≥11 kPa were used as cutoffs for the presence of minimal fibrosis (F0-1), significant fibrosis (F≥2), advanced fibrosis (F≥3), and cirrhosis (F=4), respectively. We classified the patients into two groups, namely, a minimal fibrosis group (F0-1) and a significant fibrosis group (F2-4), based on the different treatment strategies for these patients.

### 2.3. Clinical and Laboratory Data

Patient data collected retrospectively from medical records for description as well as comparison between VCTE-classified groups of severities of liver fibrosis were patient demographics and baseline characteristics including body mass index (BMI), serum albumin (g/dL), total bilirubin (mg/dL), serum creatinine (mg/dL), HBeAg serostatus, HBV DNA level (IU/mL), aspartate transaminase (AST) level (U/L), alanine transaminase (ALT) level (U/L), alkaline phosphatase (ALP) level (U/L), international normalized ratio (INR), white blood cell count (WBC) (/mm3), hemoglobin (g/dL), and platelet (x109/L). Determinants of significant liver fibrosis in chronic hepatitis B patients for multivariable logistic regression analysis were gender (reference, female gender), BMI, comorbidity of diabetes mellitus, AST (reference, ULN and unit of AST independent variable, n-fold ULN), ALT (reference, ULN and unit of ALT independent variable, n-fold ULN), ALP (U/L), and hemoglobin (g/dL). BMI was categorized as normal (BMI <23 kg/m2), overweight (BMI ≥23 kg/m2 and <25 kg/m2), or obese (BMI ≥25 kg/m2) according to the World Health Organization guidelines for adult Asian population [17]. The ULN for AST and ALT were both defined as 40 U/L, and a normal range of INR was defined as 0.80-1.10. Serum samples were tested for the presence of hepatitis B surface antigen (HBsAg), HBeAg, and anti-HBeAg antibody using radioimmunoassay (Abbott Laboratories, Irving, TX). Serum HBV viral load was measured using real-time HBV assays by polymerase chain reaction using the COBAS® AmpliPrep/COBAS® TaqMan® HBV Test, (Roche Molecular Diagnostics, Indianapolis, IN) with the range from 20 - 1.7 x 108 IU/mL.

### 2.4. Statistical Analysis

Data analysis was performed using SPSS software version 17.0 (SPSS Inc., Chicago, IL). Descriptive statistics were presented a mean (SD) or median (range and IQR) as appropriate for continuous data as well as numbers and prevalences for categorical data. Categorical variables were compared using the Chi-square test or Fisher exact test as appropriate. Continuous variables were compared for significant differences between multiple groups using one-way ANOVA test or Kruskal-Wallis test as appropriate. Multivariable logistic regression analysis was used to identify the determinants of liver fibrosis and cirrhosis, and results were presented as adjusted odds ratios (aOR) with 95% confidence intervals. A p-value <0.05 was considered statistically significant.

## 3. Results

Two hundred twenty-nine Thai chronic hepatitis B patients were identified and assessed for eligibility. Three patients (1.3%) with invalid VCTE results and 20 patients (9.6%) with missing laboratory data were excluded. The remaining 206 patients were included, and none of them had received any antiviral therapy. The median liver stiffness was 5.50 kPa (range 2.50-63.90; IQR 1.40-24.60). Of the 206 included patients, 120 patients (58.3%) were female, and 86 patients (41.7%) were male. The mean age of patients was 50 years (SD 12.4), and 32.5% were obese (BMI ≥25 kg/m2). The prevalence of hepatitis B e antigen negative (HBeAg -ve) was 83%. The median serum HBV viral load was 4340 IU/mL (range 20 - 271883036). VCTE results, demographics, and laboratory data of the patients are summarized in Table 1.

The prevalences of liver fibrosis defined by VCTE were minimal fibrosis (F 0-1) in 74.3%, significant fibrosis (F2) in 5.34%, advanced fibrosis (F3) in 11.7%, and cirrhosis (F4) in 8.74% (Table 2).

Comparison of the baseline characteristics of the patients with minimal fibrosis (F0-1) and significant fibrosis (F2-4) are shown in Table 3. A higher proportion of male patients had significant fibrosis compared with minimal fibrosis (64.2 versus 34.0%, P <0.001). BMI was significantly higher in patients with significant fibrosis than minimal fibrosis (25.30 [SD 4.64] versus 23.50 [SD 3.49] kg/m^2^, P = 0.004). Diabetes mellitus was observed significantly less frequently in patients with minimal fibrosis than significant fibrosis (5.23 versus 17.0%, P = 0.007). Both HBeAg status and serum HBV viral load were not significantly different among patients with different stages of liver fibrosis. Laboratory data showed significantly higher levels of ALP, WBC, hemoglobin, and higher prevalences of AST and ALT level >1 x ULN in the significant fibrosis group than the minimal fibrosis group (P <0.05).

Univariate analysis showed determinants for significant liver fibrosis (F2-4) were male gender, BMI, comorbidity with diabetes mellitus, hemoglobin, ALP, and high AST and ALT levels (>1 x ULN). Multivariable logistic regression analysis, however, showed that only male gender and a high AST level (>1 x ULN) were significant determinants of significant liver fibrosis (F2-4) with an aOR of 3.24 (95%CI: 1.36-7.72) and 5.71 (95%CI: 2.03-16.04), respectively (Table 4).

## 4. Discussion

The present study demonstrated a prevalence of liver fibrosis in Thai chronic hepatitis B patients defined by VCTE, a validated noninvasive test for assessment of liver fibrosis. The prevalences were minimal fibrosis (F0-1) in 74.3%, significant fibrosis (F2) in 5.34%, advanced fibrosis (F3) in 11.7%, and cirrhosis (F4) in 8.74%. Around one-quarter of these patients thus had significant liver disease (F2-4), requiring further evaluation for specific treatment for hepatitis B virus infection.

Male gender appears to hasten the progression in HBV-related liver disease. The male-to-female ratio increased from 1.2 in asymptomatic carriers to 6.3 in chronic liver disease and finally to 9.8 in hepatocellular carcinoma [18]. This gender disparity was experimentally confirmed in a HBV-transgenic-mice model. Wang et al. study demonstrated the androgen hormone could increase the transcription of HBV through direct binding to the androgen-responsive element sites in viral enhancer I. The adult male mice consequently showed higher serum titers of both viral DNA and HBsAg than the female mice [19]. This may explain the increased risk of liver-related disease in human males compared with females. We found similar results in the present study from multivariate analysis, in which male gender was a significant determinant of significant liver fibrosis (F2-4) with an aOR of 3.24 (95%CI: 1.36-7.72).

Obesity leads to adverse health consequences, including diabetes mellitus and atherosclerosis, and it contributes to the development of hepatic steatosis, nonalcoholic fatty liver disease and nonalcoholic steatohepatitis [20]. Lee et al. studied the impact of BMI on liver histology in chronic HBeAg -ve patients, and they found that overweight (BMI ≥23 kg/m^2^) contributed to hepatic necroinflammation and obesity (BMI ≥25 kg/m^2^) led to hepatic fibrosis [21]. Diabetes mellitus has also been reported to be associated with more severe liver fibrosis and cirrhosis in chronic hepatitis B patients [22]. From our data, both factors seemed to be associated with significant liver fibrosis in the univariate analysis, but were not significant in the multivariable analysis. These results might be explained by the difference of patient characteristics between the present study and Lee et al.'s study. That study had a higher HBV DNA viral load compared with our study (1.09 x 10^6^ versus 4.34 x 10^3^ IU/mL, respectively), and this factor potentiates more severe liver disease. The lower prevalence of diabetes mellitus in the present study (8.3%) compared with that of Papatheodoridis et al.'s study (14.4%)[22] might explain our the lack of significant association of diabetes mellitus with significant liver disease in multivariable analysis.

Elevations in AST level greater than ALT have been associated with more advanced fibrosis [23, 24] and are partially related to delayed clearance of AST relative to ALT [25] or to mitochondrial injury associated with more advanced fibrosis [26]. Our finding that a high AST level was, therefore, associated with more advanced fibrosis, is consistent with previous studies [27, 28]. The present study showed that patients with an AST levels of >1 x ULN have a higher rate of significant fibrosis compared with minimal fibrosis (45.3 versus 7.2%, respectively; p <0.001). From the multivariable analysis, a high AST level of >1 x ULN was associated with significant fibrosis with an aOR of 5.71 (95%CI: 2.03-16.04). This simple surrogate biomarker of liver disease, which is routinely collected in the follow-up visits by our chronic hepatitis B patients, is thus valuable because it is strongly suggestive of high risk for significant liver disease. This positive finding should cause the doctor to have a low threshold for further evaluation by either VCTE or liver biopsy to define the underlying liver fibrosis status.

Our study had several limitations including the retrospective study design, the limited number of patients, the diagnosis of liver fibrosis, and cirrhosis not based on liver biopsy, which is still the gold standard. There was also a rather low prevalence of significant fibrosis in the present study compared with a previous study [8] (25.7 versus 50.3%). This might be explained by selection bias. This is because some patients who already were diagnosed as cirrhosis by ultrasonography may not have had VCTE performed and were thus not included. Finally, as this study represents patients already referred to a tertiary care center, referral bias was always a possibility, by which patients with persistently normal ALT and low viremia are less likely to be referred.

In conclusion, our study demonstrated that a small but significant proportion of Thai chronic hepatitis B patients (about 25%) had significant liver fibrosis (F2-4). Male patients with an AST level >1 x ULN were especially at high risk of significant liver disease. VCTE or liver biopsy should therefore be performed in these patients to evaluate the status of liver fibrosis and provide an indication for antiviral treatment to prevent the development of end-stage liver disease.

## Figures and Tables

**Table 1:** Demographic and laboratory data of patients.

**Variable**	**Value**
Total patients with a valid transient elastography scan, n	206
Liver stiffness, median (range), kPa	5.50 (2.50-63.90)
Interquartile range, median (range)	11.00 (1.40-24.60)
Failed scan, n (%)	3 (1.3)
Success rate, %	98.7
Treatment naïve patients, n (%)	206 (100.0)
Age, mean (SD), y	50.01 (12.39)
Gender, n (%)	
Female	120 (58.3)
Male	86 (41.7)
BMI, mean (SD), kg/m^2^	23.95 (3.88)
<23, n (%)	94 (45.6)
≥23, n (%)	45 (21.9)
≥25, n (%)	67 (32.5)
Diabetes mellitus, n (%)	17 (8.3)
HBeAg, n (%)	
Positive	35 (17.0)
Negative	171 (83.0)
HBV DNA, median (range), IU/mL	4340 (20-271883036)
Biochemical markers	
Total bilirubin, median (range), mg/dL	0.58 (0.19-3.53)
AST, median (range), U/L	26 (8-199)
≤1 x ULN, n (%)	171 (83.0)
>1 x ULN, n (%)	35 (17.0)
ALT, median (range), U/L	25 (5-166)
≤1 x ULN, n (%)	155 (75.2)
>1 x ULN, n (%)	51 (24.8)
ALP, median (range), U/L	65 (29-511)
Albumin, median (range), g/dL	4.40 (2.20-5.40)
INR, median (range)	1.00 (0.90-2.40)
WBC, median (range), /mm^3^	6500 (2700-18900)
Hemoglobin, mean (SD), g/dL	13.13 (1.58)
Platelet, mean (SD), x10^9^/L	220.00 (63.60)
Creatinine, mean (SD), mg/dL	1.14 (1.61)

kPa: Kilopascal; BMI: Body mass index; HBeAg: Hepatitis B e antigen;

AST: Aspartate aminotransferase; ALT: Alanine aminotransferase; ULN: Upper limit of normal;

ALP: Alkaline phosphatase; INR: International normalized ratio.

**Table 2:** Prevalence of liver fibrosis by vibration-controlled transient elastography in chronic hepatitis B patients.

**Liver fibrosis stage**	**Overall ** **n (**%**)**
F0-1	153 (74.3)
F2	11 (5.3)
F3	24 (11.7)
F4	18 (8.7)

**Table 3:** Comparison of the baseline characteristics of patients with minimal fibrosis (F0-1) and significant fibrosis (F2-4).

**Variable**	**F0-1 ** **(n = 153)**	**F2-4 ** **(n = 53)**	**P-value**
Age, mean (SD), y	49.60 (12.73)	51.21 (11.39)	0.417
Male gender, n (%)	52 (34.0)	34 (64.2)	<0.001
BMI, mean (SD), kg/m^2^	23.50 (3.49)	25.30 (4.64)	0.004
<23, n (%)	75 (49.0)	19 (35.8)	
≥23, n (%)	34 (22.2)	11 (20.8)	
≥25, n (%)	44 (28.8)	23 (43.4)	
Diabetes mellitus, n (%)	8 (5.2)	9 (17.0)	0.007
HBeAg, n (%)			0.998
Positive	26 (17.0)	9 (17.0)	
Negative	127 (83.0)	44 (83.0)	
HBV DNA level, n (%)			0.395
<2000 IU/mL	68 (44.4)	20 (37.7)	
≥2000 IU/mL	85 (55.6)	33 (62.3)	
Total bilirubin, mean (SD), mg/dL	0.60 (0.25)	0.75 (0.54)	0.053
AST level, n (%)			<0.001
≤1 x ULN	142 (92.8)	29 (54.7)	
>1 x ULN	11 (7.2)	24 (45.3)	
ALT level, n (%)			<0.001
≤1 x ULN	130 (85.0)	25 (47.2)	
>1 x ULN	23 (15.0)	28 (52.8)	
ALP, median (range), U/L	63 (29-280)	73 (32-511)	0.011
Albumin, mean (SD), g/dL	4.40 (0.40)	4.30 (0.40)	0.134
INR, mean (SD)	1.00 (0.10)	1.10 (0.20)	0.051
WBC, median (range), /mm^3^	6800 (2700-18900)	8200 (3800-11100)	0.036
Hemoglobin, mean (SD), g/dL	13.00 (1.54)	13.50 (1.63)	0.044
Platelet, mean (SD), x10^9^/L	239.00 (60.50)	221.00 (70.90)	0.082

BMI: Body mass index; HBeAg: Hepatitis B e antigen;

AST: Aspartate aminotransferase; ALT: Alanine aminotransferase; ULN: Upper limit of normal;

ALP: Alkaline phosphatase; INR: International normalized ratio.

**Table 4:** Multivariable logistic regression analysis on factors associated with significant fibrosis (F2-4).

**Variable**	**Crude OR (95**%**CI)**	**P-value**	**Adjusted OR (95**%**CI)**	**P-value**
Gender (Ref=female)	3.48(1.81-6.68)	<0.001	3.24(1.36-7.72)	0.008
Body mass index (kg/m^2^)	1.12(1.03-1.21)	0.006	1.03(0.92-1.14)	0.650
Diabetes mellitus	3.71(1.35-10.18)	0.011	2.32(0.72-7.49)	0.160
AST (Ref=≤1 x ULN)	10.68(4.72-24.20)	<0.001	5.71(2.03-16.04)	0.001
ALT (Ref=≤1 x ULN)	6.33(3.15-12.72)	<0.001	1.90(0.75-4.84)	0.178
ALP (U/L)	1.02(1.01-1.03)	0.004	1.01(1.00-1.02)	0.520
Hemoglobin (g/dL)	1.24(1.00-1.54)	0.046	0.97(0.72-1.29)	0.825

AST: Aspartate aminotransferase; ALT: Alanine aminotransferase; ULN: Upper limit of normal;

ALP: Alkaline phosphatase; OR: Odds ratio; 95%CI: Confidence interval 95%.

## Data Availability

Raw data were generated at Division of Gastroenterology, Department of Medicine, Rajavithi Hospital, College of Medicine, Rangsit University, Bangkok, Thailand. Derived data supporting the findings of this study are available from the corresponding author on request.

## Additional Points

*Study Highlights*. VCTE have recently been increasingly used in clinical practice to evaluate liver fibrosis stage instead of liver biopsy. Recent guidelines from the EASL-ALEH recommends VCTE as a valid noninvasive test for assessing liver fibrosis in HBV-infected patients. By VCTE we demonstrated that the prevalence of significant liver fibrosis in Thai chronic hepatitis B patients was 25.7% and determinants of significant liver fibrosis were male gender and high AST level.
